# Can carbon emissions from tropical deforestation drop by 50% in 5 years?

**DOI:** 10.1111/gcb.13153

**Published:** 2016-02-09

**Authors:** Daniel J. Zarin, Nancy L. Harris, Alessandro Baccini, Dmitry Aksenov, Matthew C. Hansen, Claudia Azevedo‐Ramos, Tasso Azevedo, Belinda A. Margono, Ane C. Alencar, Chris Gabris, Adrienne Allegretti, Peter Potapov, Mary Farina, Wayne S. Walker, Varada S. Shevade, Tatiana V. Loboda, Svetlana Turubanova, Alexandra Tyukavina

**Affiliations:** ^1^ Climate and Land Use Alliance235 Montgomery Street13th FloorSan FranciscoCA94104USA; ^2^ Forests ProgramWorld Resources Institute10 G Street NEWashingtonDC20002USA; ^3^ The Woods Hole Research Center149 Woods Hole RoadFalmouthMA02540USA; ^4^ Transparent WorldRossolimo str, 5/22, Building 1MoscowRussia; ^5^ Department of Geographical SciencesUniversity of MarylandCollege ParkMD20742USA; ^6^ Núcleo de Altos Estudos AmazônicosUniversidade Federal do Para‐UFPAAv. Perimetral, No. 1, GuamaBelémPará66075‐750Brazil; ^7^ Observatório do ClimaRua Deputado Lacerda Franco144 ap 181CEP 05418‐000São PauloSPBrazil; ^8^ Direktorat General of Climate ChangeMinistry of Environment and Forestry of IndonesiaManggala Wanabhakti 7th block 12th floorJl Gatot SubrotoJakarta10270Indonesia; ^9^ Instituto de Pesquisa Ambiental da AmazoniaSHIN CA 5, Bloco J2 – Sala 309Lago NorteDF 71503‐505Brazil; ^10^ Blue Raster2200 Wilson Blvd., Suite 210ArlingtonVA22201USA

**Keywords:** Brazil, carbon emissions, deforestation, forests, Indonesia, New York Declaration on Forests

## Abstract

Halving carbon emissions from tropical deforestation by 2020 could help bring the international community closer to the agreed goal of <2 degree increase in global average temperature change and is consistent with a target set last year by the governments, corporations, indigenous peoples' organizations and non‐governmental organizations that signed the New York Declaration on Forests (NYDF). We assemble and refine a robust dataset to establish a 2001–2013 benchmark for average annual carbon emissions from gross tropical deforestation at 2.270 Gt CO_2_ yr^−1^. Brazil did not sign the NYDF, yet from 2001 to 2013, Brazil ranks first for both carbon emissions from gross tropical deforestation and reductions in those emissions – its share of the total declined from a peak of 69% in 2003 to a low of 20% in 2012. Indonesia, an NYDF signatory, is the second highest emitter, peaking in 2012 at 0.362 Gt CO_2_ yr^−1^ before declining to 0.205 Gt CO_2_ yr^−1^ in 2013. The other 14 NYDF tropical country signatories were responsible for a combined average of 0.317 Gt CO_2_ yr^−1^, while the other 86 tropical country non‐signatories were responsible for a combined average of 0.688 Gt CO_2_ yr^−1^. We outline two scenarios for achieving the 50% emission reduction target by 2020, both emphasizing the critical role of Brazil and the need to reverse the trends of increasing carbon emissions from gross tropical deforestation in many other tropical countries that, from 2001 to 2013, have largely offset Brazil's reductions. Achieving the target will therefore be challenging, even though it is in the self‐interest of the international community. Conserving rather than cutting down tropical forests requires shifting economic development away from a dependence on natural resource depletion toward recognition of the dependence of human societies on the natural capital that tropical forests represent and the goods and services they provide.

## Introduction

Over 180 governments, companies, indigenous peoples' organizations, and non‐governmental organizations have signed the New York Declaration on Forests (NYDF), launched at the United Nations Climate Summit in September 2014 (UN Climate Summit, 2014). The NYDF represents a shared agenda of its signatories that grew in large part from a confluence of interests in advancing the implementation of (1) intergovernmental partnerships to reduce emissions from deforestation and forest degradation, and foster conservation, sustainable management of forests, and enhancement of forest carbon stocks (REDD+) that emerged within the United Nations Framework Convention on Climate Change (UNFCCC); (2) corporate commitments to remove deforestation from agricultural commodity supply chains that have been made by large‐scale purchasers, traders, and producers of those commodities in response to advocacy campaigns that have influenced consumer‐facing brands; and (3) land and resource tenure reform efforts that aim to formalize the rights of indigenous and other traditional communities to forests that they have generally sought to protect from encroachment.

Within the context of this shared vision, the NYDF identifies a number of global targets, including

At least halve the rate of loss of natural forests globally by 2020 and strive to end natural forest loss by 2030.

But the NYDF, though accompanied by a number of discrete action plans, neither provided a benchmark against which success would be evaluated nor specified how its targets would be realized. Here, we bring together new and robust global and national data to set a benchmark for halving carbon emissions from gross tropical deforestation and propose where reductions will likely need to occur if that target is to be met. The 2030 target, along with the other NYDF targets, is beyond the purview of our analysis.

We limit our scope to the area within the tropical latitudes because most recent conversion of natural forest to a new land use (i.e. deforestation, FAO‐FRA, 2000) occurs within the tropics. We emphasize halving carbon emissions from tropical deforestation rather than halving its area (but see Fig. S1 for a comparative analysis) because climate change is so clearly the context for the NYDF agenda and REDD+ is essential to meeting the <2 degree threshold that the international community has adopted. We specify reducing ‘gross’ deforestation (the loss in naturally forested area caused by conversion of forest cover to non‐forested land) rather than ‘net’ deforestation (the net change in the area of forested land) because the impact of the former on carbon emissions is unambiguous, whereas the latter equates young, low‐carbon forest growth with old, high‐carbon forest loss (Brown & Zarin, 2013).

The annual rate of carbon emissions from gross deforestation is calculated by multiplying an estimate of the area of gross deforestation and an estimate of the aboveground carbon content of that area. The rate of natural forest loss referenced in the NYDF is directly related only to the area term; hence, our emphasis on halving carbon emissions from gross tropical deforestation is consistent with the NYDF target, but not identical. Our area‐based analysis in the Appendix S1 suggests that halving the area of gross deforestation may require a more diffuse distribution of the reductions across geographies than halving carbon emissions from gross deforestation, because more carbon‐dense tropical forests are concentrated in a smaller subset of countries.

## A global benchmark for carbon emissions from gross tropical deforestation

In this section, we provide a description of the datasets we have compiled and modified to provide our best estimates for benchmarking carbon emissions from gross tropical deforestation. We recognize that there is no scientific consensus on a global dataset for gross deforestation rates or associated carbon emissions and that, with few exceptions, lack of consensus applies at national level as well. We fully expect our estimates to be improved upon and hope that we contribute to the acceleration of such improvements by making our data compilations fully and freely available on the Global Forest Watch Climate website (climate.globalforestwatch.org).

Edenhofer *et al*. (2014) present a synthesis of global net CO_2_ flux estimates from deforestation and reforestation/regrowth, and there are a wide range of data sources that include different processes, definitions, and approaches to calculating these fluxes (Ramankutty *et al*., 2007; Houghton *et al*., 2012; Le Quéré *et al*., 2013; Pongratz *et al*., 2014). The United Nations Food and Agricultural Organization's Forest Resource Assessment (e.g. FAO‐FRA 2015) is a generally cited source of data on global deforestation but is not well‐suited to our purpose because its estimates are based on country self‐reporting that lacks the spatial detail needed to adequately integrate deforestation area and carbon content, and because it focuses on net forest cover change rather than gross deforestation (Grainger, 2008).

Recent estimates of carbon loss from tropical deforestation derived from carbon stock and forest area loss data vary from 0.81 to 2.9 Pg annually (Pan *et al*., 2011; Baccini *et al*., 2012; Harris *et al*., 2012; Achard *et al*., 2014; Tyukavina *et al*., 2015), with the greatest differences found between studies that use earth observation satellite data and those that use forest inventory and other tabular reference data. Annual data derived from earth observation satellites, available for the years 2001–2014, constitute the most up to date, globally consistent observations of tree cover loss and gain (Hansen *et al*., 2013 and updates available at globalforestwatch.org). An important caveat regarding the Hansen dataset, however, is that the definition of gross tree cover loss includes rotational clear‐felling of management units within tree plantations and clearing of forest regrowth in shifting cultivation cycles, along with conversion of natural forests. We took steps that address this issue for four countries that are substantial contributors to total carbon emissions from gross tropical deforestation – Brazil, Indonesia, Democratic Republic of the Congo (DRC), and Malaysia (see below and in the Appendix S1).

**Figure 1:**
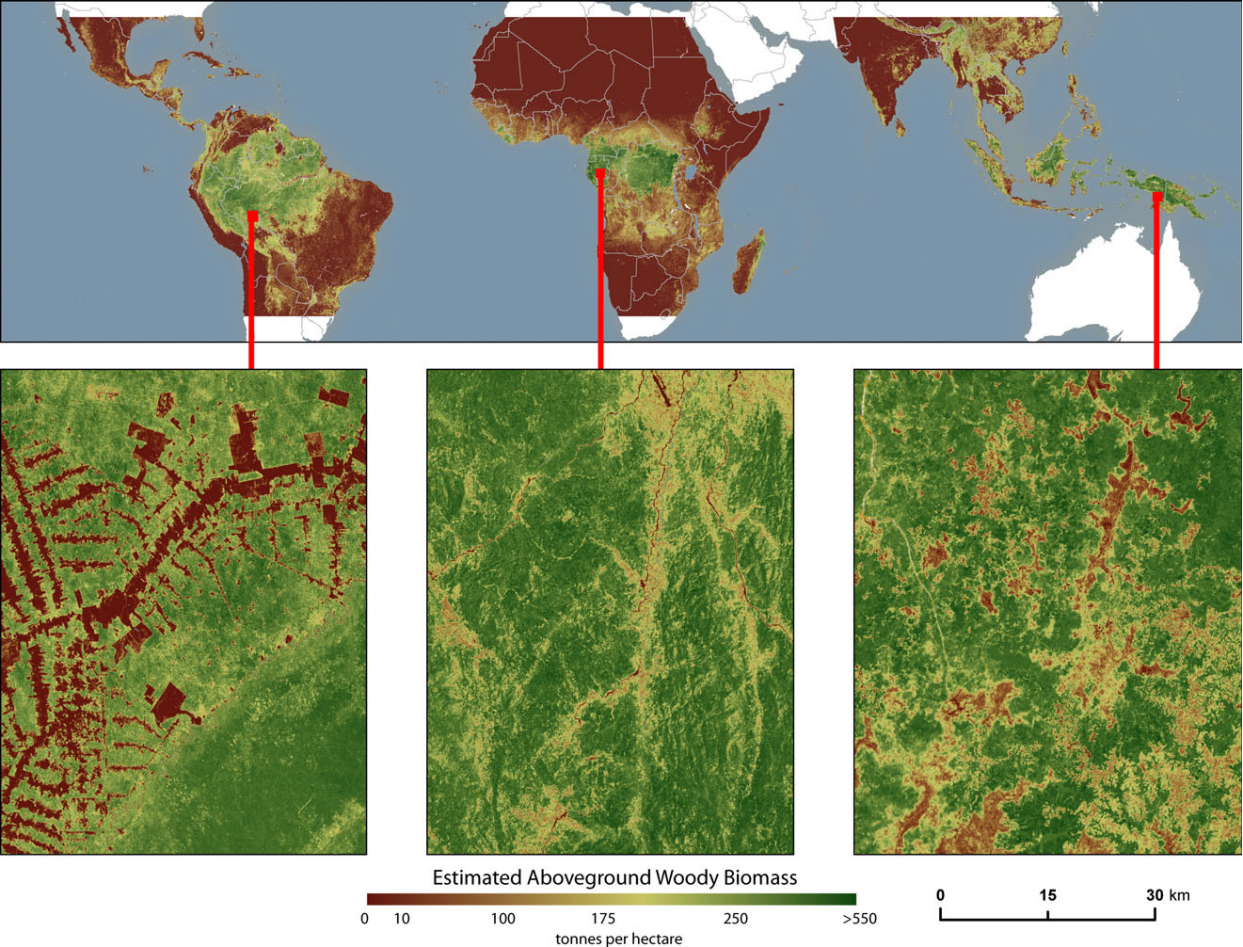
Pantropical map of aboveground live woody biomass density (Mg biomass ha^−1^) at 30 m resolution for circa the year 2000, with insets depicting different patterns of deforestation occurring in tropical America, tropical Africa, and tropical Asia.

Pantropical maps of aboveground biomass density (Saatchi *et al*., 2011; Baccini *et al*., 2012) have established baseline estimates of forest carbon stocks. Our analysis expands upon the methodology presented in Baccini *et al*. (2012) to generate a pan‐tropical map of aboveground live woody biomass density at 30 m resolution for circa the year 2000 (Fig. 1). This new map allows for the first time the co‐location of biomass estimates with tree cover loss estimates at similar spatial resolution. We used the statistical relationship derived between ground‐based measurements of forest biomass density and colocated Geoscience Laser Altimeter System (GLAS) LiDAR waveform metrics as described by Baccini *et al*. (2012) to estimate the biomass density of more than 40 000 GLAS footprints throughout the tropics. Then, using randomForest models (Breiman, 2001), the GLAS‐derived estimates of biomass density were correlated to continuous, gridded variables including Landsat 7 ETM+ satellite imagery and products (e.g. reflectance), elevation, and biophysical variables (Baccini *et al*., 2004). By using continuous, gridded datasets as inputs to the randomForest models, this work yielded a wall‐to‐wall 30‐m resolution map of aboveground woody biomass density across the tropics, now available at climate.globalforestwatch.org. A more detailed description of the methodology used to create this data product is included in the Appendix S1.

We established a benchmark estimate of carbon emissions from tropical deforestation by combining spatial data layers of annual tree cover loss and aboveground live woody biomass density circa the year 2000. Tree cover loss estimates for a given spatial extent represent the sum of all pixels with vegetation taller than 5 m in height with greater than 25% tree cover in the year 2000 that experienced loss in tree cover as a stand‐replacement disturbance between 2001 and 2013. We used the 25% tree cover threshold for consistency, recognizing that some countries have lower thresholds for defining forested areas and that numerous threatened tropical woodland regions may fall below this threshold, despite their conservation value.

Biomass loss estimates for a given spatial extent represent the sum of all aboveground biomass contained within loss pixels prior to clearing, as of the year 2000. We converted aboveground biomass loss to aboveground carbon loss using a conversion factor of 0.5 (Penman *et al*., 2003) and carbon to carbon dioxide using a conversion factor of 3.67. We consider all of the aboveground carbon as ‘committed’ emissions to the atmosphere upon deforestation (Ramankutty *et al*., 2007; Houghton *et al*., 2012) although there are lag times associated with some aboveground carbon pools.

Other carbon pools such as belowground biomass and soil carbon are excluded from this analysis but we recognize that emissions from those pools can be substantial, especially for organic soils (e.g. in southeast Asian peatlands (Couwenberg *et al*., 2010; Krug *et al*., 2013). As this year's fires in Indonesia indicate, clearing and draining of peatlands to convert them to fiber and palm oil plantations creates a readily ignitable emissions source that, when the rainy season is delayed, can rapidly reach over a billion tons of CO_2_‐e (Global Fire Emissions Database 2015). In the absence of fire, emissions from peatland soil degradation have been estimated at 355–855 Mt CO_2_ yr^−1^, including emissions from new drainage and ongoing emissions as drained peat decomposes (Hooijer *et al*., 2010). The nature and dynamics of peatland soil emissions are sufficiently distinct from aboveground carbon loss and characterized by larger and different sources of uncertainty in their quantification and accounting that we consider them beyond our scope here.

For Brazil, Indonesia, DRC, Malaysia, Colombia, Ecuador, Guyana, and Mexico, we replaced or supplemented all or part of the global tree cover loss and woody biomass datasets with national‐level data, as described below. These eight countries include those with the largest share of carbon emissions from gross tropical deforestation, and cumulatively account for two‐thirds of the 2001–2013 emissions benchmark.

### Brazil

Data for Brazil (Table S1) are from the System of Greenhouse Gas Emissions Estimates (SEEG), maintained by the Climate Observatory to provide annual estimates of greenhouse gas (GHG) emissions across all sectors, including land use, land use change, and forestry, on a consistent and accessible basis (SEEG, 2014). GHG estimates are generated according to Intergovernmental Panel on Climate Change guidelines, based on data from the Second Brazilian Inventory of Anthropogenic GHG Emissions and Removals, elaborated by Brazil's Ministry of Science, Technology and Innovation, supplemented by data gathered from government reports, institutes, research centers, sectoral groups, and non‐governmental organizations. Emission estimates are derived by multiplying an estimate of the area of gross deforestation by an estimate of aboveground carbon content. The SEEG estimates cover GHG emissions over the period 1970–2013 for all sectors, except deforestation, which covers the period 1990–2013. SEEG uses first‐order estimates for deforestation, which commit 100% of emissions at the time of conversion, consistent with our approach for other countries. For the Amazon biome, SEEG deforestation estimates are derived from the annual monitoring conducted by the National Institute for Space Research (Prodes/INPE). For the Atlantic Forest (Mata Atlantica), the data are also detailed and frequent, but not annual. Information is provided by the Ministry of Environment (MMA) and the Atlas of Forest Remnants of the Atlantic, conducted by the SOS Atlantic Forest Foundation with INPE images. For other biomes – Cerrado, Caatinga, Pantanal and Pampa – data are also from MMA and not annual. Average carbon stocks in forest aboveground biomass for SEEG emission estimates are derived from RADAMBRASIL forest inventories, based on a classification map of vegetation types of the Brazilian Institute of Geography and Statistics and is the same information used in the national inventory (RADAMBRASIL 1981).

### Indonesia

Indonesia has a relatively high proportion of tree cover loss associated with plantation harvests. This type of loss is included in the Hansen dataset but excluded from a more recent analysis of loss within Indonesia's primary forests (Margono *et al*., 2014). In the context of the NYDF target of halving the rate of natural forest loss globally by 2020, we used the primary forest dataset (Margono *et al*., 2014) to define forest area in Indonesia in the year 2000. Primary forests were defined as all mature forests of 5 ha or more in extent that retain their natural composition and structure and have not been completely cleared in recent history (i.e. at least 30 years in age). Spatial distribution of primary forest as defined by Margono *et al*. (2014) showed 90.2% agreement with the Indonesian Ministry of Forestry's primary forest map for the year 2000. Biomass estimates within the primary forest mask area were derived from the global biomass density map described above. Applying the primary forest mask for Indonesia reduced the estimate of gross deforestation from 1.31 to 0.52 Mha yr^−1^ with a corresponding emissions reduction from 0.394 to 0.198 Gt CO_2_ yr^−1^ (Table S2).

### Democratic Republic of the Congo

We also used a primary forest mask for DRC to estimate emissions occurring from gross deforestation as of the year 2000 (Potapov *et al*., 2012). The primary forest class was defined by Potapov *et al*. as mature humid tropical forest with greater than 60% canopy cover. Shifting cultivation mosaics are a prevalent, long‐established land use in DRC (Mayaux *et al*., 2004; Defourny *et al*., 2011; DeWasseige *et al*., 2012; Potapov *et al*., 2012). After the initial cycle of clearing (from primary forest), cultivation, and forest regrowth (to secondary forest), subsequent cycles often include regrowth intervals that are long enough to recover pre‐clearing quantities of secondary forest aboveground biomass. In this sense, stable shifting cultivation mosaics are similar to tree plantations for area‐based carbon accounting, insofar as only the initial clearing of natural forest should be counted as gross deforestation. For DRC, our focus on primary forest aims to exclude secondary forest clearing associated with stable shifting cultivation cycles, while still capturing emissions from expansion of shifting cultivation into primary forests.

For DRC, applying the primary forest mask reduced the estimate of gross deforestation from 0.581 to 0.110 Mha yr^−1^ with a corresponding emissions reduction from 0.194 to 0.046 Gt CO_2_ yr^−1^ (Table S3).

### Malaysia

Malaysia also has a high proportion of tree cover loss associated with plantation harvests; therefore, including these areas would substantially inflate gross deforestation emission estimates. Unlike Indonesia and DRC, no primary forest mask layer is currently available for Malaysia. Instead, we corrected for tree cover loss occurring outside of natural forests by mapping tree plantations across Malaysia (including peninsular Malaysia, Sabah, and Sarawak) as of the year 2013/2014 using visual interpretation of Landsat imagery supplemented with high‐resolution satellite imagery, field data, and other ancillary georeferenced information. In some cases, multiple image dates before the target year were required to detect and verify plantation presence. Unlike a single scene, the time series allowed the visualization of seasonal changes and long‐term dynamics which are often critically important for the identification and delineation of tree plantations. Landsat imagery was supplemented with visual checks of high‐resolution satellite data, online crowdsourcing services containing descriptions or photos, and field trips to check the presence of plantations and accuracy of contours. The resulting map for peninsular Malaysia was assessed for accuracy in an independent double‐blind study using a stratified random sample of 807 of an original 1000 non‐adjacent points (500 points within and 500 points outside plantations). Some points were discarded due to the presence of cloud cover. These points served as ‘ground‐truth’ information from which a standardized set of statistics (i.e. confusion matrix) could be generated. Recent high‐resolution imagery from Digital Globe (2013–2015) was used as the ‘ground‐truth’ dataset. Overall accuracy of the plantation map product was 87%, with a user's accuracy of 79% and producer's accuracy of 94%. While some assessment of geometric and geographic fidelity is implicit in this method, less can be stipulated about the accuracy of the exact boundaries of plantations. The total area of mapped plantations within Malaysia, based on the 2013/14 imagery, was 9.9 Mha, with 5.68 Mha in peninsular Malaysia, 2.35 Mha in Sabah, and 2.01 Mha in Sarawak. All deforestation within that mapped plantation area was excluded from our analysis. The result of this masking process reduced the estimate of tree cover loss from 0.387 to 0.162 Mha yr^−1^ with a corresponding emissions reduction from 0.118 to 0.043 Gt CO_2_ yr^−1^ (Table S4, Fig. S2). That result is a conservative estimate insofar as it fails to account for natural forest loss in the fraction of the masked area that represents new plantations created during the 2001–2013 interval, as the mask was based on a 2013/14 map product. This is unlikely to create a substantial gap between actual and estimated emissions for peninsular Malaysia, where plantations have a longer history, but it may be significant for Sabah and Sarawak.

### Colombia, Ecuador, Guyana, Mexico

Within the context of the UNFCCC, Parties to the Convention have been requested to submit forest reference emission levels (FRELs) that establish a business‐as‐usual baseline against which monitored emissions going forward will be compared under an international initiative now known as REDD+. These FRELs are guided by several ‘modalities’ (Decision 12/CP.17 and its Annex) that state that when establishing FRELs, Parties should do so transparently taking into account historic data and adjusting for national circumstances in accordance with relevant decisions of the Parties to the UNFCCC. FRELs can be developed subnationally as an interim measure while transitioning to a national scale. A step‐wise approach is allowed that enables Parties to improve the FREL by incorporating better data, improved methodologies and, where appropriate, additional carbon pools. FRELs are expressed in units of tons of CO_2_ equivalent per year and must maintain consistency with a country's GHG inventory.

To date, six countries have submitted FRELs: Brazil, Colombia, Ecuador, Guyana, Mexico, and Malaysia. For this analysis, we do not incorporate information submitted to the UNFCCC by Brazil because, to be consistent across all countries, we use data for a more recent time period (2001–2013) and for all of Brazil, as summarized above, rather than using the 1996–2010 reference time period and covering the Amazon only as indicated in Brazil's FREL. We also do not incorporate information submitted to the UNFCCC by Malaysia because their FREL focuses on sustainable forest management rather than reducing emissions from deforestation.

For Colombia, Ecuador, Guyana, and Mexico, we downloaded the country submissions and used reported annual CO_2_ emission estimates for the stated historical reference period, years which overlap with our benchmark period of 2001–2013 (Table S5). As an interim measure, Colombia chose to submit a subnational reference level that covers only the Colombian Amazon. In this case, we used data submitted to the UNFCCC for the Colombian Amazon. Outside the Colombian Amazon, we used the Hansen data and biomass density estimates as described above. To delineate the two regions, we downloaded the geographic boundary for the Colombian Amazon submitted with Colombia's reference level. In cases where submitted reference periods do not cover the full 2001–2013 time period of our analysis, we assumed a continuation of the historical average to fill missing years.

## Reaching for the 2020 target

**Figure 2:**
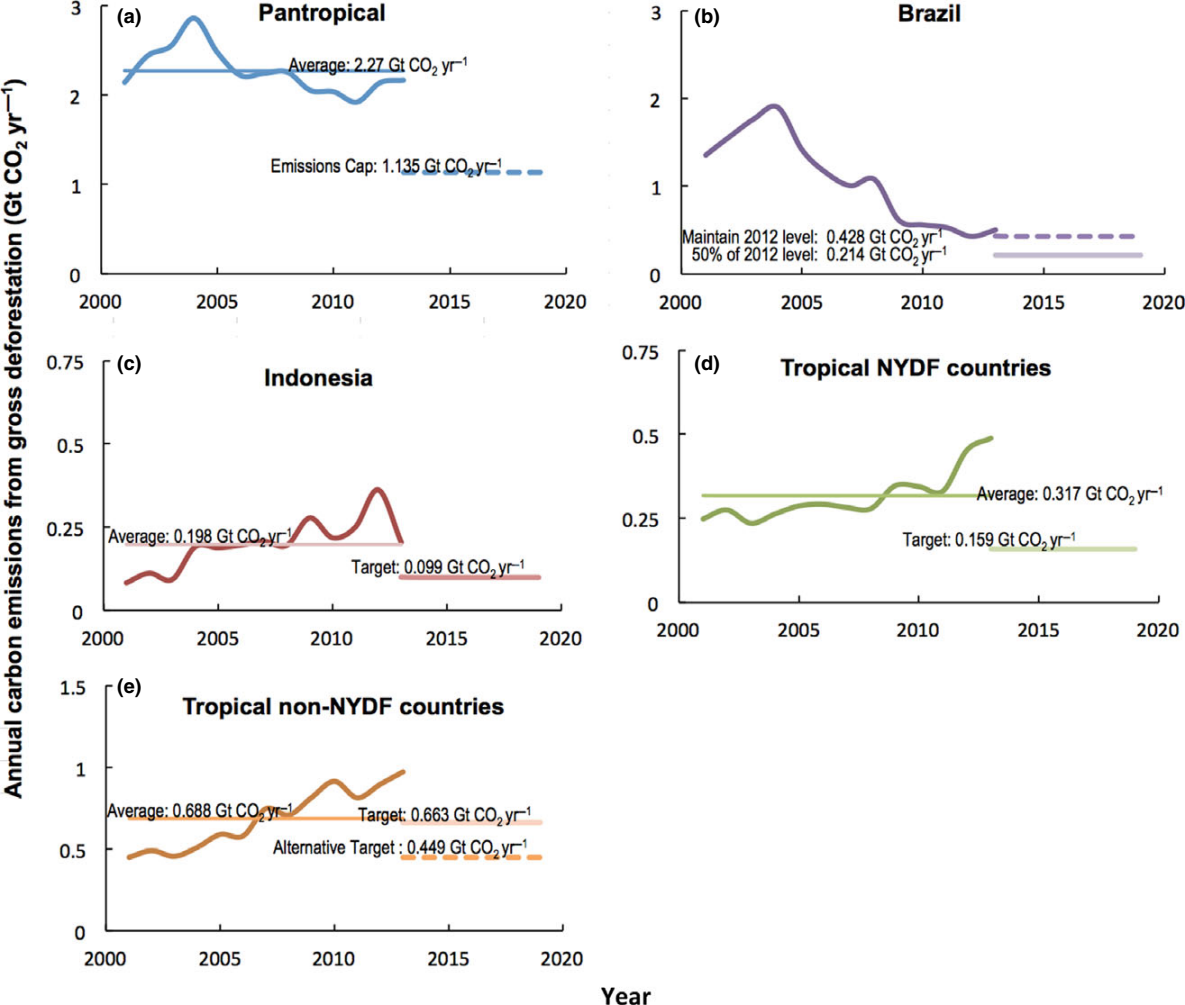
Benchmark carbon emissions from gross deforestation for (a) all countries within the pantropical study area; (b) Brazil; (c) Indonesia primary forests; (d) all tropical signatories of the New York Declaration on Forests*; and (e) remaining tropical forested countries that did not sign the Declaration. Emission caps for each country or group reflect calculations described in the text. Note scale differences on vertical axes. *Indonesia is not included in (d) although they did sign the Declaration; nor is Brazil, which did not.

Using the myriad data sources described above, we established an historical average from 2001 to 2013 for carbon emissions from gross tropical deforestation, against which we benchmark the NYDF target of a 50% reduction by 2020 (Fig. 2a). During this interval, carbon emissions from gross tropical deforestation averaged 2.270 Gt CO_2_ yr^−1^, closely matching a recent estimate derived from a stratified sampling methodology (2.165 Gt CO_2_ yr^−1^, Tyukavina *et al*., 2015), and within the range of other estimates in the published literature (see Table 4 in Tyukavina *et al*., 2015 for the comparisons). We characterize the target of cutting emissions from our benchmark in half by 2020 as an ‘emissions cap’ of 1.135 Gt CO_2_ yr^−1^ (Fig. 2a).

Our analysis extends to forests in 102 countries, but we emphasize the critical role of the top emitters because, by definition, they have the most to contrib‐ute to emission reduction. The collective ability and willingness of over a hundred countries to deliver the results necessary to achieve the halving of carbon emissions from tropical deforestation in the next 5 years hinges on both domestic and international factors.

Brazil remains the single largest source of carbon emissions from gross deforestation, despite declines, both absolute and proportional, achieved during the 2001–2013 period (Fig. 2b). Brazil is followed by Indonesia, where carbon emissions from gross deforestation increased from 2001 to 2012 and then declined sharply in 2013 (Fig. 2c). We also separate out tropical country signatories to the NYDF as a group because they have declared the common ambition of halving natural forest loss by 2020, so we assume they are correspondingly committed to reducing their own carbon emissions from gross deforestation (Fig. 2d). We then estimate the average rate of emissions from the remaining tropical forest countries over the next 5 years (Fig. 2e) that would keep the total under the pantropical cap of 1.135 Gt CO_2_ yr^−1^. Below we identify where reductions are likely needed to stay under that cap.

### Brazil

Brazil stands out not only as the largest source of carbon emissions from gross deforestation but also due to its decade‐long decline, during which constraining the frontier for agricultural expansion spurred intensification and innovation in the sector rather than impeding growth in production. In 2012, Brazil's emissions from gross deforestation accounted for just 20% of the tropical total, down considerably from a peak 69% in 2003. Carbon emissions from gross deforestation in Brazil bottomed out at 0.428 Gt CO_2_ yr^−1^ in 2012, slightly lower than the 2020 target set in the country's national climate change law. If Brazil maintained its emissions at 2012 levels, it would be responsible for 38% of the 2020 emissions cap of 1.135 Gt CO_2_ yr^−1^, leaving 0.707 Gt CO_2_ yr^−1^ available for other tropical forest countries.

Brazil's success in reducing carbon emissions from gross deforestation over the past decade is due largely to a combination of public policy initiatives, law enforcement, and voluntary actions by the private sector (Lapola *et al*., 2013). We suggest that the same combination can deliver even deeper reductions between now and 2020. Expansion of agricultural production could continue in Brazil for at least the next 25 years without further conversion of natural ecosystems (Strassburg *et al*., 2014), and further halving of the 2012 rate, perhaps to as low as 0.214 Gt CO_2_ yr^−1^, is likely to be possible by 2020.

Reducing deforestation in undesignated public lands could be one way to deliver further emission cuts. Although Brazil has higher governance capacity than most tropical nations, most of its deforestation is still illegal, despite innovative and effective command and control efforts put into place since 2005 (Arima *et al*., 2014). For example, in 2013, over 80 million hectares of undesignated public lands remained in Brazil's Amazon region. These are largely ungoverned spaces, subject to illegal land clearing. Brazil made large strides in public land designation between 2003 and 2010, and an interministerial process underway for the past 2 years could make substantial additional progress to help reduce this problem (SFB, 2013; Press Release 2015). Political threats to protected area and indigenous territory designations will also need to be addressed (e.g. http://www2.camara.leg.br/camaranoticias/noticias/DIREITO-E-JUSTICA/498534-COMISSAO-ESPECIAL-RETOMA-DISCUSSAO-DA-PEC-DAS-DEMARCACOES-DE-TERRAS-INDIGENAS.html).

Legal compliance on private properties could put a further dent in deforestation and associated emissions. The implementation of Brazil's revised Forest Code is well underway, with requirements for substantial forest conservation by landowners across the country. The Brazilian government has publicly announced their explicit aims of registering all rural properties by May 2016 and rooting out illegality. If fully implemented, 193 Mha of native vegetation in Legal Reserves and Riparian Preservation Areas would be protected, equivalent to 87 Gt CO_2_ (Soares Filho *et al*., 2014).

Finally, no‐deforestation commodity commitments by major supply chain companies have had significant success in the Brazilian Amazon – nearly halting deforestation for soy production since 2006, as well as impacting the beef sector, where their potential has only recently begun to be realized (Gibbs *et al*., 2015). Positive incentives to encourage conservation of forests that are legally vulnerable to deforestation remain largely untested in Brazil but may prove effective as well (Brazil‐German Joint Statement on Climate Change 2015). Particular attention will need to be paid to small farmers, particularly those within agrarian settlement areas, which are now responsible for a third of the ongoing deforestation in the Amazon region.

### Indonesia

We assume Indonesia is committed to halving its own carbon emissions from gross deforestation by 2020 because it signed the NYDF (Brazil did not). Relative to the 2001–2013 benchmark, that would mean reducing their average rate of emissions over the next 5 years to 0.099 Gt CO_2_ yr^−1^ (Fig. 1c). Carbon emissions from gross deforestation declined in Indonesia from a 2012 high of 0.362–0.205 Gt CO_2_ yr^−1^ in 2013. The significant downturn in emissions from deforestation in 2013 is likely due to a combination of price and policy signals, particularly in the palm oil and pulp and paper sectors, but their relative importance, and the sustainability of that downturn, remains uncertain. That uncertainty is highlighted by the catastrophic fires occurring this year across the Indonesian archipelago, emitting what may amount to more than ten times the amount of CO_2_ than the 2001–2013 average annual gross deforestation benchmark (Global Fire Emissions Database, 2015).

Although the major drivers of deforestation in Indonesia – plantation expansion for palm oil and for pulp and paper production – are increasingly covered by voluntary ‘no‐deforestation’ commitments made by the supply chain companies that dominate Indonesia's palm oil and pulp and paper sectors, implementation to date has been mixed, and so has the Indonesian government's response to these commitments (Seymour, 2015). Reducing emissions from gross deforestation to 0.099 Gt CO_2_ yr^−1^ would likely be consistent with full implementation of the no‐deforestation commitments. Austin *et al*. (2015) project that compliance with those commitments could reduce emissions from oil palm expansion by up to 60%. Indonesia's President extended a moratorium on the issuance of new deforestation licenses earlier this year, and recently announced that all peatland development should immediately cease while all existing licenses on peatland are reviewed and that his government will implement a major peatland restoration effort, including blocking drainage canals, to ensure that the conditions that allow for catastrophic fires do not arise in the future (Seymour, 2015; official transcript at http://setkab.go.id/pengantar-presiden-joko-widodo-pada-rapat-pengendalian-bencana-kabut-asap-di-kantor-presiden-jakarta-23-oktober-2015/).

### NYDF signatories

**Figure 3:**
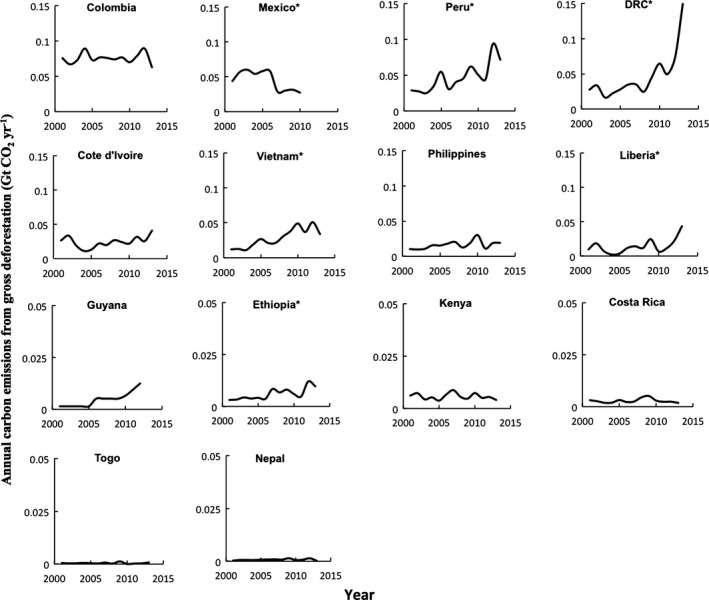
Trajectory of carbon emissions from deforestation between 2001 and 2013 from tropical country signatories of the New York Declaration on Forests (for Indonesia, see Fig. 2). Carbon emissions in the Democratic Republic of the Congo represent those from primary forests only. Countries with an * have an emissions profile that trended significantly over the time period (*P *<* *0.05). Note differences in vertical axis scale among countries.

Figure 2d illustrates the 13‐year emissions trajectory for the other 14 tropical NYDF signatory countries. NYDF countries (excluding Indonesia) were responsible for 12% of carbon emissions from gross tropical deforestation in 2001, but as a group grew to 23% in 2013, with emissions accelerating at an annual rate of approximately 16.5 Mt CO_2_ yr^−2^ (*R* ^2^ = 0.74, Fig. 2d). Disaggregating the 14 tropical countries included in this group (Fig. 3) indicates that most of this trend is due to recent increases in DRC and Peru, with upward trending also significant for Vietnam and Liberia. While noting that this group's emissions rose from 2001 to 2013, we infer that their shared commitment will include at least halving their collective carbon emissions from gross deforestation from a historical average of 0.317 down to 0.159 Gt CO_2_ yr^−1^, but thus far only Mexico exhibits a statistically significant downward trend (Fig. 3). Many of the NYDF countries have also signed the Lima Challenge (UN, 2015), in which they shared their intent to come forward with significant voluntary pledges to reduce deforestation emissions using domestic resources, and to do more if adequate international finance is made available. Meanwhile, the German, Norwegian, and British governments have announced their own commitment to finance up to 20 new REDD+ programs, pending receipt of proposals from developing countries that they deem robust and credible (Press Release 2014).

### Other tropical forest countries

**Figure 4:**
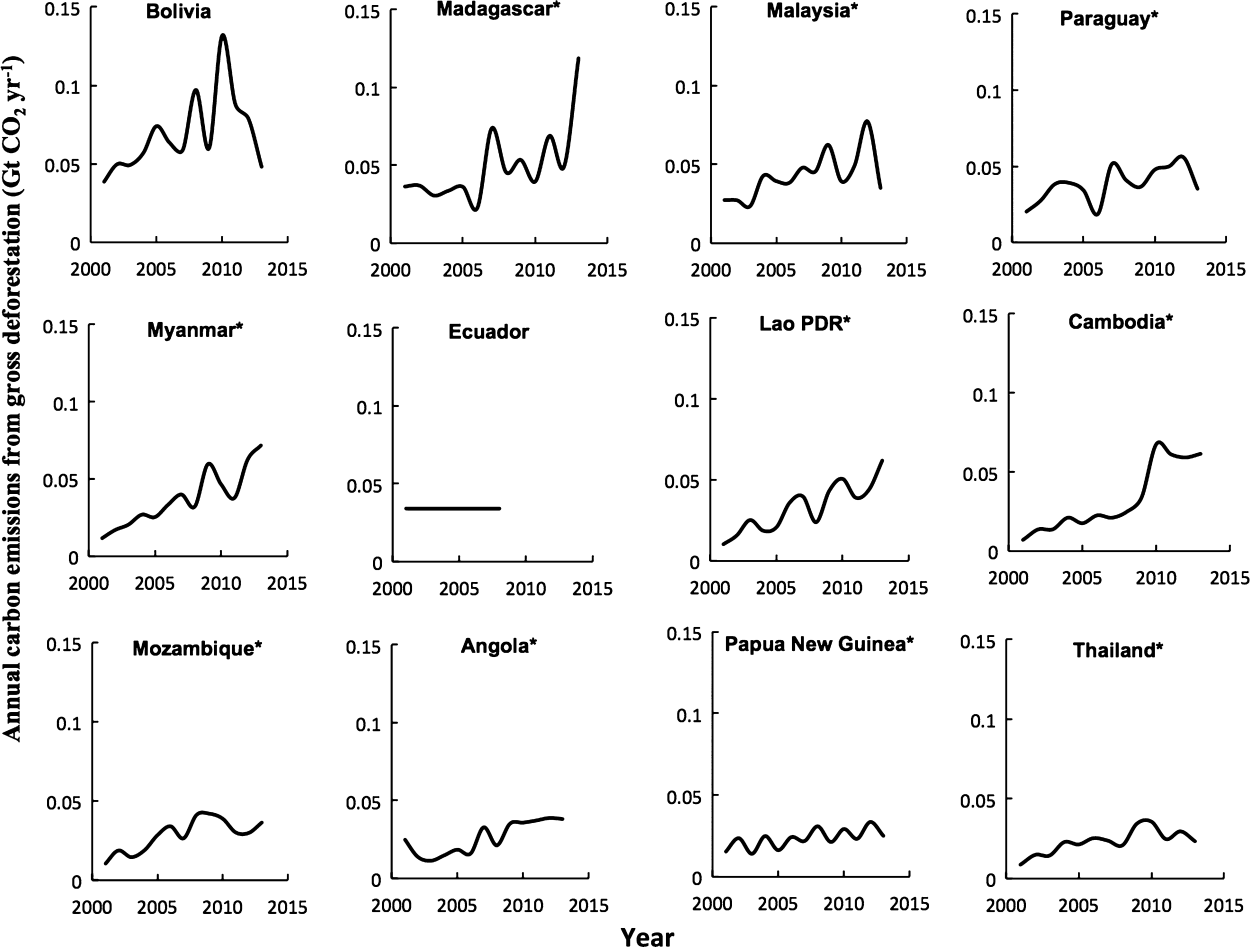
Trajectory of top tropical forest emitters that did not sign the New York Declaration on Forests. Countries with an * have an emissions profile that trended significantly over the time period (*P *<* *0.05). Emissions for Ecuador reflect the 8‐year historical average as reported in their forest reference emission level to the United Nations Framework Convention on Climate Change.

The 2001–2013 historical average of these 86 countries was 0.688 Gt CO_2_ yr^−1^ (Fig. 2e) and exhibited a significant upward trend during this interval (*R* ^2^ = 0.93, Fig. 2e). The top dozen emitters in this group – Bolivia, Madagascar, Malaysia, Paraguay, Myanmar, Ecuador, Laos, Cambodia, Mozambique, Angola, Papua New Guinea, and Thailand – were responsible for 64% of the emissions of the group during the benchmark period (Fig. 4). By 2013, many of these countries were already emitting twice their 2001–2013 average.

Overall, tropical forest countries could stay within the 2020 gross deforestation emissions cap of 1.135 Gt CO_2_ yr^−1^ in 2020 if Brazil maintained its 2012 level of 0.428 Gt CO_2_ yr^−1^, Indonesia and the other NYDF countries reduced by half to 0.099 and 0.159 Gt CO_2_ yr^−1^, respectively, and the remaining 86 tropical forest countries reduced by 35% to 0.449 Gt CO_2_ yr^−1^. Alternatively, if Brazil delivered the extra 0.214 Gt CO_2_ yr^−1^ reduction we believe can be achieved – by further reducing illegal deforestation on both public and private lands, maintaining and building upon the private sector's existing no‐deforestation commodity commitments, and piloting positive incentives, including for small farmers – then the 2020 target could be met as long as Indonesia and the NYDF countries delivered their 50% cut, and the other tropical forest countries that did not sign the NYDF collectively reduced their average emissions by just 4% (Fig. 2e).

Even that 4% reduction should not be seen as trivial. When we disaggregate the global data over the benchmark period, the general trend indicates that large declines in carbon emissions from gross deforestation in Brazil have now been offset by increases in many other countries (Fig. 2d, e). While Brazil and Indonesia continue to be the largest emitters from gross deforestation, their proportional responsibility is a shrinking fraction of the global total. Recognition of this trend is reflected in the number of countries now participating in various REDD+ finance mechanisms, including those administered by the World Bank and the United Nations, and the commitment of leading donors to increase their support (e.g. Press Release 2014).

## Conclusions

The two scenarios we propose for achieving targeted reductions in carbon emissions from gross tropical deforestation are summarized in Table 1. These are intended to be illustrative; a hybrid, as well as other options, may also be possible. Significantly, Brazil is critical to any scenario, even though it did not sign the NYDF. And the combination of public policy reforms, law enforcement, and voluntary private sector actions responsible for Brazil's success thus far are likely to prove broadly applicable elsewhere, although the details will surely vary according to national circumstances.

**Table 1:** Two potential scenarios by which tropical countries can achieve a target of halving carbon emissions from gross tropical deforestation in the next 5 years. Values within columns may not sum due to rounding errors

Country/group	Average carbon emissions 2001–2013 (Gt CO_2_ yr^−1^)	Emission caps to achieve 2020 target (Gt CO_2_ yr^−1^)	
		Scenario 1 (Brazil maintains emissions at 2012 levels)	Scenario 2 (Brazil achieves additional reductions)
Brazil	1.066	0.428	0.214
Indonesia	0.198	0.099	0.099
NYDF signatories	0.317	0.159	0.159
Other countries	0.688	0.449	0.663
Pantropical	2.270	1.135	1.135

A decade ago, the deforestation reductions that Brazil has already achieved were unimaginable. Today, cutting carbon emissions from gross tropical deforestation in half in the next 5 years, both in Brazil and globally, may seem just as unlikely, even with the benefit of Brazil's recent experience.

With expectations building for the upcoming UNFCCC Conference of Parties in Paris (e.g. Kolbert, 2015), the size of this challenge and the transition it implies should not be underestimated. Conserving rather than cutting down tropical forests requires shifting economic development away from a dependence on natural resource depletion toward recognition of the dependence of human societies on the natural capital that tropical forests represent, and the goods and services they provide (TEEB, 2010; Francis, 2015). To the extent that dependence extends beyond national borders to provide global public benefits such as climate change mitigation, it is in the self‐interest of the international community to promote and support the leadership of tropical forest countries that undertake the transition. And to the extent that the sustainability of commerce is also at stake, it is similarly in the self‐interest of the private sector both to support and to lead.

## Supporting information


**Appendix S1.** Supplemental analysis, methodological detail, and data.
**Figure S1.** Benchmark gross deforestation rates for (a) all countries within the pantropical study area; (b) Brazil; (c) Indonesia primary forests; (d) all tropical signatories of the New York Declaration on Forests; and (e) remaining tropical forested countries that did not sign the Declaration.
**Figure S2.** Mapped plantations and tree cover loss in Malaysia between 2001‐2013 for (a) peninsular Malaysia, (b) Sabah and (c) Sarawak.
**Table S1.** Gross deforestation and carbon emission estimates for Brazil. Source: System of Greenhouse Gas Emissions Estimates (SEEG).
**Table S2.** Tree cover loss and carbon emission estimates for areas inside and outside primary forests of Indonesia.
**Table S3.** Tree cover loss and carbon emission estimates for areas inside and outside primary forests of the Democratic Republic of Congo.
**Table S4.** Tree cover loss and carbon emissions from deforestation estimated for areas inside and outside plantation boundaries mapped for the year 2014.
**Table S5.** Reference level information submitted by Parties to the UNFCCC and used in this analysis as national data.
**Table S6.** Deforestation and carbon emissions from deforestation for Colombia, Ecuador, Guyana and Mexico.
