# Supplementary material for: Can carbon emissions from tropical deforestation drop by 50% in 5 years?
Source: Glob Chang Biol. 2016 Feb 9;22(4):1336–47. doi: 10.1111/gcb.13153 (PMC5021154; doi:10.1111/gcb.13153)
Supplement: Supplementary file 1 — Appendix S1. Supplemental analysis, methodological detail, and data. Figure S1. Benchmark gross deforestation rates for (a) all countries within the pantropical study area; (b) Brazil; (c) Indonesia primary forests; (d) all tropical signatories of the New York Declaration on Forests; and (e) remaining tropical forested countries that did not sign the Declaration. Figure S2. Mapped plantations and tree cover loss in Malaysia between 2001‐2013 for (a) peninsular Malaysia, (b) Sabah and (c) Sarawak. Table S1. Gross deforestation and carbon emission estimates for Brazil. Source: System of Greenhouse Gas Emissions Estimates (SEEG). Table S2. Tree cover loss and carbon emission estimates for areas inside and outside primary forests of Indonesia. Table S3. Tree cover loss and carbon emission estimates for areas inside and outside primary forests of the Democratic Republic of Congo. Table S4. Tree cover loss and carbon emissions from deforestation estimated for areas inside and outside plantation boundaries mapped for the year 2014. Table S5. Reference level information submitted by Parties to the UNFCCC and used in this analysis as national data. Table S6. Deforestation and carbon emissions from deforestation for Colombia, Ecuador, Guyana and Mexico. [file GCB-22-1336-s001.docx]

**Supporting Information for “Can carbon emissions from tropical deforestation drop by 50% in five years?”**

**Halving carbon emissions vs. halving deforestation rates**

The NYDF focuses explicitly on halving the rate of natural forest loss (i.e., deforestation) rather than on halving carbon emissions. In the main text, we highlighted carbon emissions to focus on climate change mitigation, and outline how this reduction could be distributed among tropical countries. We also conducted a similar analysis on the basis of area and concluded that achieving an area-based target could be more difficult to achieve than a carbon-based target. Cutting in half the 13-year historical average pantropical deforestation rate of 7.36 Mha yr^-1^ would translate to a tropical deforestation cap of 3.68 Mha yr^-1^ in 2020 (Fig S1a). If Brazil maintained its 2012 deforestation rate of 1.49 Mha yr^-1^ (Fig S1b), Indonesia cut its deforestation in half compared to 2013 levels (0.52 Mha yr^-1^ in 2013 to 0.26 Mha yr^-1^ by 2020, Fig S1c), and tropical NYDF signatories fulfilled their commitments to cut their average deforestation rates in half (1.02 Mha yr^-1^ in 2013 to 0.51 Mha yr^-1^ by 2020, Fig S1d), then these countries collectively would be responsible for 62% of the calculated 2020 cap of 3.68 Mha yr^-1^, leaving a deforestation cap for the remaining 85 countries of 1.42 Mha yr^-1^ in 2020, or up to 2.17 Mha yr^-1^ if Brazil makes the extra reduction (i.e. from implementing additional governance and no-deforestation commodities). The 2001-2013 historical average of those 86 countries is 2.80 Mha yr^-1^ (Fig S1e), so they would collectively need to reduce their average deforestation rates by 22% to 49% if the 2020 area target is to be met. Achieving the 50% reduction in the area of gross tropical deforestation would require a more diffuse distribution than the 50% reduction in emissions from gross tropical deforestation, due to the relative concentration of higher carbon-density forests in a limited number of countries.


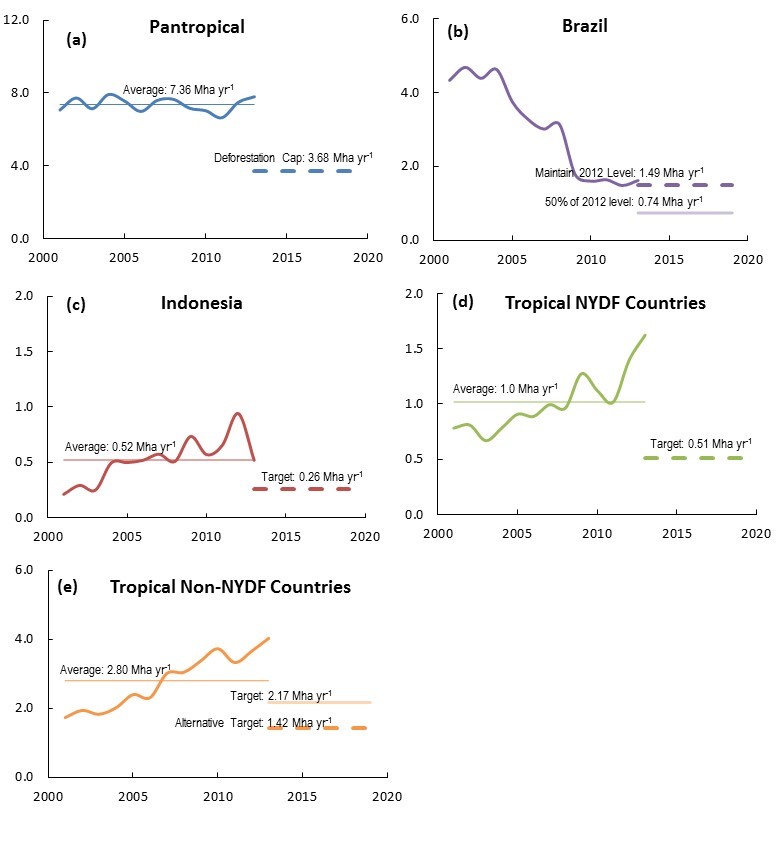


Figure S1. Benchmark gross deforestation rates for (a) all countries within the pantropical study area; (b) Brazil; (c) Indonesia primary forests; (d) all tropical signatories of the New York Declaration on Forests^[[1]](#footnote-1)^; and (e) remaining tropical forested countries that did not sign the Declaration. Deforestation caps, or targets, for each country or group reflect calculations in the text. Note scale differences on vertical axes.

**Year**

**Biomass Estimation Procedure**

This section provides a more detailed description of the methodology used to generate the pan-tropical map of aboveground live woody biomass density at 30 meter resolution for ca. year 2000. To generate this dataset, we followed the approach developed by Baccini *et al.* (2012), which uses field-based biomass measurements; co-located LiDAR waveform metrics from the Geoscience Laser Altimeter System (GLAS) instrument; and MODIS NADIR, BRDF-Adjusted Reflectance (NBAR; Schaaf *et al.* 2002). Baccini *et al.* (2012) combined these datasets in a multistep modeling procedure, yielding a pan-tropical map of carbon density at 500 meter resolution. In the current analysis, we followed a similar modeling procedure and used the same field to GLAS statistical relationship developed in Baccini et al 2012. To create a 30 m resolution map, we replaced the MODIS data with Landsat satellite imagery and biophysical variables and elevation.

The first modeling step, which remains unchanged in this analysis, is detailed in Baccini et al. 2012. Ground-based measurements of tree stem diameter at breast height were collected as part of a large-scale field campaign throughout the tropics. The tree stem diameter measurements were collected within 40 m by 40 m square plots centered within GLAS LiDAR footprints (circular footprints with 70 m diameter) and converted to biomass estimates using allometric equations from Chave *et al.* (2005). By linking estimates of ground-based biomass density estimates with co-located GLAS LiDAR data, we developed a multiple regression equation that predicts biomass density based on GLAS LiDAR waveform metrics. This multiple regression equation was then applied to roughly 40,000 GLAS LiDAR footprints across the tropics, yielding an estimate of biomass for each LiDAR footprint.

In the second stage of the modeling procedure, we developed statistical models that relate GLAS-based biomass estimates to spatially continuous datasets. More specifically for this new work, we replaced the MODIS data used in Baccini et al. (2012) with Landsat satellite imagery and trained new RandomForest models to predict aboveground biomass density based on Landsat satellite imagery, biophysical variables and elevation. The Landsat datasets included in our new work include reflectance in the red band, near-infrared band, and two shortwave-infrared bands (corresponding to bands 3, 4, 5, and 7), the Normalized Difference Vegetation Index (NDVI), the Normalized Difference Infrared Index (NDII), and percent of tree canopy cover from the Hansen et al. (2013) Global Forest Change dataset. Additional datasets used as predictors in the RandomForest models include 3-arc second SRTM elevation data and gridded biophysical data at 1 km resolution, resampled at 30 meters to match with the reflectance data (Hijmans et al. 2005). Among the biophysical predictor variables, continental models used precipitation, maximum temperature, temperature seasonality, the minimum temperature of the coldest month, the temperature annual range, annual precipitation, precipitation of the driest quarter, and precipitation of the coldest quarter.

We trained a separate RandomForest model for each continent - tropical America, Africa, and Asia - using a stratified sampling design of roughly 13,000 GLAS-based biomass estimates for each model. The models explained about 72%, 58%, and 66% of the variance in biomass density in America, Africa, and Asia, respectively. By training the random forest models with spatially continuous, pan-tropical predictors, this work yields a wall-to-wall 30 m map of aboveground woody biomass density across the tropics.

**National Data Used for Benchmark Estimates**

Table S1.Gross deforestation and carbon emission estimates for Brazil. Source: System of Greenhouse Gas Emissions Estimates (SEEG).

|  | **Annual Gross Deforestation, ha yr^-1^** | | | | | | | | | | | | |
| --- | --- | --- | --- | --- | --- | --- | --- | --- | --- | --- | --- | --- | --- |
| **Biome** | **2001** | **2002** | **2003** | **2004** | **2005** | **2006** | **2007** | **2008** | **2009** | **2010** | **2011** | **2012** | **2013** |
| Amazonia | 1,816,500 | 2,165,100 | 2,539,600 | 2,777,200 | 1,901,400 | 1,428,600 | 1,165,100 | 1,291,100 | 746,400 | 700,000 | 641,800 | 457,100 | 589,100 |
| Cerrado | 1,569,775 | 1,569,775 | 1,417,900 | 1,417,900 | 1,417,900 | 1,417,900 | 1,417,900 | 1,417,900 | 763,700 | 646,900 | 741,500 | 765,300 | 765,300 |
| Caatinga | 590,459 | 590,459 | 276,267 | 276,267 | 276,267 | 276,267 | 276,267 | 276,267 | 192,100 | 192,100 | 192,100 | 192,100 | 192,100 |
| Mata Atlantica | 261,689 | 261,689 | 45,700 | 45,700 | 45,700 | 45,700 | 45,700 | 45,700 | 24,800 | 15,183 | 14,090 | 21,977 | 23,948 |
| Pampa | 205 | 205 | 36,383 | 36,383 | 36,383 | 36,383 | 36,383 | 36,383 | 33,100 | 33,100 | 33,100 | 33,100 | 33,100 |
| Pantanal | 96,154 | 96,154 | 71,327 | 71,327 | 71,327 | 71,327 | 71,327 | 71,327 | 18,850 | 18,850 | 18,850 | 18,850 | 18,850 |
| **Total** | **4,334,781** | **4,683,381** | **4,387,177** | **4,624,77** | **3,748,977** | **3,276,177** | **3,012,677** | **3,138,677** | **1,778,950** | **1,606,133** | **1,641,440** | **1,488,427** | **1,622,398** |
|  | **Annual Carbon Emissions from Gross Deforestation, Gt CO_2_ yr^-1^** | | | | | | | | | | | | |
| Biome | **2001** | **2002** | **2003** | **2004** | **2005** | **2006** | **2007** | **2008** | **2009** | **2010** | **2011** | **2012** | **2013** |
| Amazonia | 1.004 | 1.197 | 1.404 | 1.535 | 1.051 | 0.790 | 0.644 | 0.714 | 0.413 | 0.387 | 0.355 | 0.253 | 0.326 |
| Cerrado | 0.221 | 0.221 | 0.296 | 0.296 | 0.296 | 0.296 | 0.296 | 0.296 | 0.159 | 0.135 | 0.135 | 0.135 | 0.135 |
| Caatinga | 0.042 | 0.042 | 0.020 | 0.020 | 0.020 | 0.020 | 0.020 | 0.020 | 0.014 | 0.014 | 0.014 | 0.014 | 0.014 |
| Mata Atlantica | 0.075 | 0.075 | 0.016 | 0.016 | 0.016 | 0.016 | 0.016 | 0.016 | 0.009 | 0.005 | 0.005 | 0.008 | 0.008 |
| Pampa | 0.0001 | 0.017 | 0.017 | 0.017 | 0.017 | 0.017 | 0.017 | 0.017 | 0.016 | 0.016 | 0.016 | 0.016 | 0.016 |
| Pantanal | 0.015 | 0.013 | 0.013 | 0.013 | 0.013 | 0.013 | 0.013 | 0.013 | 0.003 | 0.003 | 0.003 | 0.003 | 0.003 |
| **Total** | **1.356** | **1.564** | **1.766** | **1.897** | **1.413** | **1.152** | **1.006** | **1.076** | **0.614** | **0.560** | **0.528** | **0.428** | **0.502** |

Table S2. Tree cover loss and carbon emission estimates for areas inside and outside primary forests of Indonesia. For this analysis, benchmark carbon emissions from deforestation were assumed to be those occurring only within primary forests.

|  | **2001** | **2002** | **2003** | **2004** | **2005** | **2006** | **2007** | **2008** | **2009** | **2010** | **2011** | **2012** | **2013** | **Average** |
| --- | --- | --- | --- | --- | --- | --- | --- | --- | --- | --- | --- | --- | --- | --- |
| **Annual tree cover loss in Indonesia vs. annual gross deforestation within Indonesia’s primary forests (ha yr^-1^)** | | | | | | | | | | | | | | |
| All tree cover loss | 727,890 | 857,338 | 549,687 | 1,291,702 | 1,175,388 | 1,439,372 | 1,387,506 | 1,393,408 | 1,951,410 | 1,283,102 | 1,544,192 | 2,269,515 | 1,162,796 | **1,310,254** |
| Deforestation within primary forests | 213,656 | 293,786 | 251,483 | 497,372 | 499,257 | 520,241 | 575,053 | 509,737 | 735,112 | 571,250 | 656,929 | 941,610 | 520,461 | **521,996** |
| **Annual carbon emissions from tree cover loss in Indonesia vs. from gross deforestation within Indonesia’s primary forests (Gt CO_2_ yr^-1^)** | | | | | | | | | | | | | | |
| All tree cover loss | 0.227 | 0.260 | 0.171 | 0.395 | 0.359 | 0.425 | 0.400 | 0.416 | 0.577 | 0.396 | 0.472 | 0.676 | 0.353 | **0.394** |
| Deforestation within primary forests | 0.083 | 0.112 | 0.093 | 0.191 | 0.188 | 0.196 | 0.208 | 0.195 | 0.278 | 0.218 | 0.250 | 0.362 | 0.205 | **0.198** |

Table S3. Tree cover loss and carbon emission estimates for areas inside and outside primary forests of the Democratic Republic of Congo. For this analysis, benchmark carbon emissions from deforestation were assumed to be those occurring only within primary forests.

|  | **2001** | **2002** | **2003** | **2004** | **2005** | **2006** | **2007** | **2008** | **2009** | **2010** | **2011** | **2012** | **2013** | **Average** |
| --- | --- | --- | --- | --- | --- | --- | --- | --- | --- | --- | --- | --- | --- | --- |
| **Annual tree cover loss in DRC vs. annual gross deforestation within DRC’s primary forests (ha yr^-1^)** | | | | | | | | | | | | | | |
| All tree cover loss | 493,305 | 559,311 | 303,428 | 432,407 | 528,037 | 491,763 | 517,012 | 436,524 | 704,404 | 883,000 | 483,875 | 704,150 | 1,017,161 | **581,106** |
| Deforestation within primary forests | 65,750 | 78,772 | 38,563 | 53,271 | 68,211 | 85,476 | 81,012 | 59,832 | 105,468 | 157,227 | 114,847 | 172,986 | 354,932 | **110,488** |
| **Annual carbon emissions from tree cover loss in DRC vs. from gross deforestation within DRC’s primary forests (Gt CO_2_ yr^-1^)** | | | | | | | | | | | | | | |
| All tree cover loss | 0.171 | 0.190 | 0.097 | 0.145 | 0.176 | 0.167 | 0.176 | 0.143 | 0.239 | 0.289 | 0.163 | 0.231 | 0.337 | **0.194** |
| Deforestation within primary forests | 0.028 | 0.033 | 0.016 | 0.022 | 0.028 | 0.035 | 0.035 | 0.025 | 0.045 | 0.065 | 0.049 | 0.073 | 0.150 | **0.046** |

Table S4. Tree cover loss and carbon emissions from deforestation estimated for areas inside and outside plantation boundaries mapped for the year 2014. For this analysis, carbon emissions from deforestation were assumed to be those occurring only outside of plantation boundaries.

|  | **2001** | **2002** | **2003** | **2004** | **2005** | **2006** | **2007** | **2008** | **2009** | **2010** | **2011** | **2012** | **2013** | **Average** |
| --- | --- | --- | --- | --- | --- | --- | --- | --- | --- | --- | --- | --- | --- | --- |
| **Annual tree cover loss in Malaysia vs. annual gross deforestation outside plantations (ha yr^-1^)** | | | | | | | | | | | | | | |
| All tree cover loss | 312,377 | 300,718 | 178,260 | 341,388 | 366,384 | 327,821 | 397,977 | 361,750 | 613,572 | 422,231 | 456,067 | 620,899 | 332,195 | **387,049** |
| Inside plantations | 228,024 | 218,444 | 111,086 | 218,337 | 251,710 | 219,139 | 262,550 | 234,735 | 431,075 | 306,784 | 319,004 | 406,415 | 226,137 | **224,649** |
| Outside plantations | 84,352 | 82,275 | 67,174 | 123,051 | 114,673 | 108,682 | 135,426 | 127,015 | 182,497 | 115,447 | 137,063 | 214,483 | 106,058 | **162,400** |
| **Annual carbon emissions from tree cover loss in Malaysia vs. from gross deforestation outside plantations (Gt CO_2_ yr^-1^)** | | | | | | | | | | | | | | |
| All tree cover loss | 0.089 | 0.088 | 0.055 | 0.104 | 0.110 | 0.102 | 0.122 | 0.114 | 0.187 | 0.129 | 0.143 | 0.197 | 0.099 | **0.118** |
| Inside plantations | 0.062 | 0.061 | 0.031 | 0.062 | 0.071 | 0.063 | 0.074 | 0.068 | 0.125 | 0.090 | 0.094 | 0.119 | 0.064 | **0.076** |
| Outside plantations | 0.027 | 0.027 | 0.024 | 0.043 | 0.039 | 0.038 | 0.048 | 0.046 | 0.062 | 0.039 | 0.049 | 0.077 | 0.035 | **0.043** |

Table S5. Reference level information submitted by Parties to the UNFCCC and used in this analysis as national data.

| **Country** | **Geographic Coverage** | **Reference Time Period** | **Data Source for deforestation estimates** | **Data Source for aboveground forest biomass estimates** |
| --- | --- | --- | --- | --- |
| Colombia | Subnational (Colombian Amazon only) | 2000-2012 | Landsat, biennial monitoring | 721 plots collected between 1990 and 2014 |
| Ecuador | National | 1990-2000, 2000-2008 | Landsat, ASTER, change estimated from comparing land cover maps | National forest inventory plots collected between 2012 and 2014 |
| Guyana | National | 2001-2012 | Landsat (up to 2010), RapidEye (2011 onwards), gross deforestation estimated. | 66 plots collected between 2012 and 2014. Four 0.1 ha subplots per plot. |
| Mexico | National | 2002/2003, 2007/2008, 2012/2013 | Landsat, SPOT, change estimated from comparing land cover maps | 21,811 systematically distributed national inventory plots collected between 2004 and 2007. Four 0.04 ha subplots per plot. |

Table S6. Deforestation and carbon emissions from deforestation for Colombia, Ecuador, Guyana and Mexico. Values in italics represent the replacement of missing values in the 2001-2013 time series (no country data reported) with average values across the time period.

| **Country** | **2001** | **2002** | **2003** | **2004** | **2005** | **2006** | **2007** | **2008** | **2009** | **2010** | **2011** | **2012** | **2013** | **Average** |
| --- | --- | --- | --- | --- | --- | --- | --- | --- | --- | --- | --- | --- | --- | --- |
| **Annual gross deforestation (ha yr^-1^)** | | | | | | | | | | | | | | |
| Guyana | 1,921 | 1,921 | 1,921 | 1,921 | 1,921 | 5,314 | 5,314 | 5,314 | 5,314 | 8,089 | 9,000 | 15,088 | *4,997* | **4,997** |
| Mexico | *(Not reported)* | | | | | | | | | | | | | |
| Colombian Amazon | 77,042 | 77,042 | 95,846 | 95,846 | 82,448 | 82,448 | 78,898 | 78,898 | 69,355 | 69,355 | 93,604 | 93,604 | *82,882* | **82,882** |
| Ecuador | 89,493 | 89,493 | 89,493 | 89,493 | 89,493 | 89,493 | 89,493 | 89,493 | *89,493* | *89,493* | *89,493* | *89,493* | *89,493* | **89,493** |
| **Annual carbon emissions from gross deforestation (Gt CO_2_ yr^-1^)** | | | | | | | | | | | | | | |
| Guyana | 0.0014 | 0.0014 | 0.0014 | 0.0014 | 0.0014 | 0.005 | 0.005 | 0.005 | 0.005 | 0.006 | 0.009 | 0.0125 | *0.0047* | **0.0047** |
| Mexico | 0.044 | 0.057 | 0.060 | 0.0054 | 0.058 | 0.058 | 0.029 | 0.030 | 0.031 | 0.027 | *0.045* | *0.045* | *0.045* | **0.045** |
| Colombian Amazon | 0.044 | 0.004 | 0.054 | 0.054 | 0.047 | 0.047 | 0.045 | 0.045 | 0.039 | 0.039 | 0.053 | 0.053 | *0.047* | **0.047** |
| Ecuador | 0.034 | 0.034 | 0.034 | 0.034 | 0.034 | 0.034 | 0.034 | 0.034 | *0.034* | *0.034* | *0.034* | *0.034* | *0.034* | **0.034** |


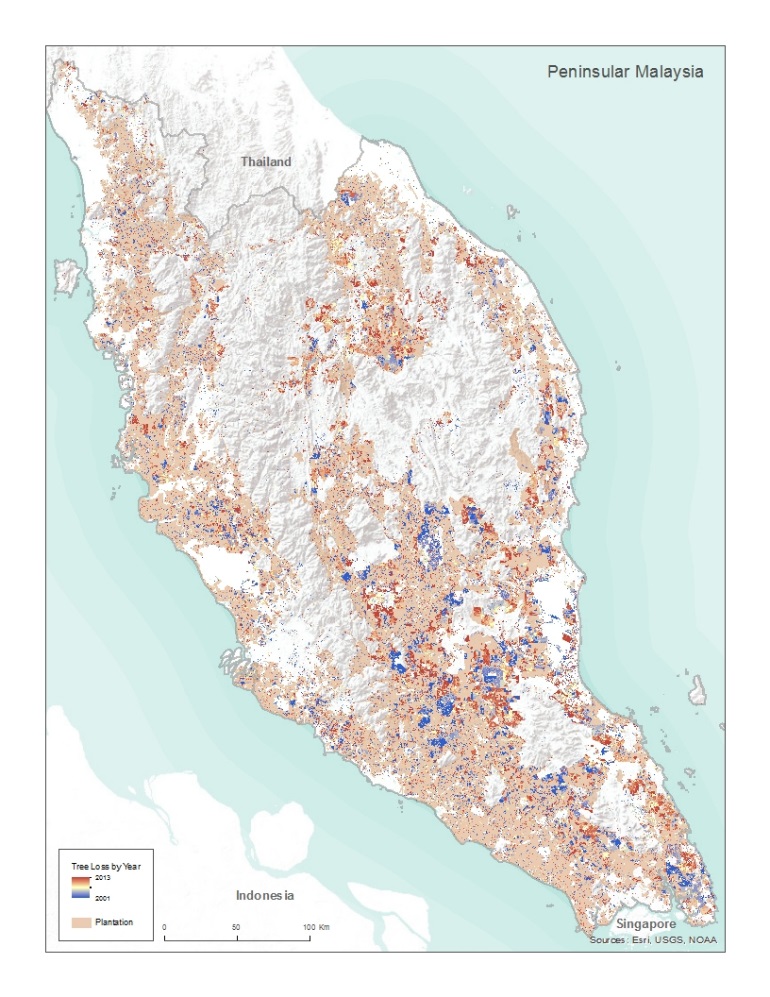

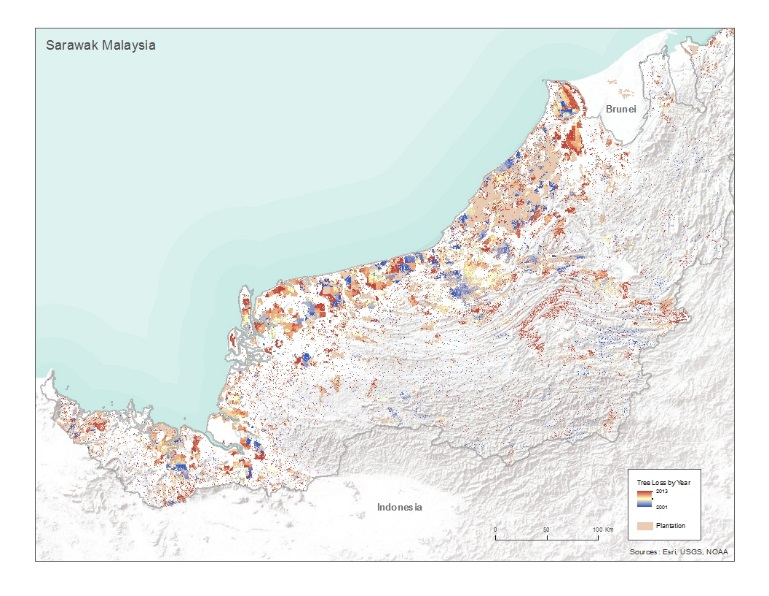

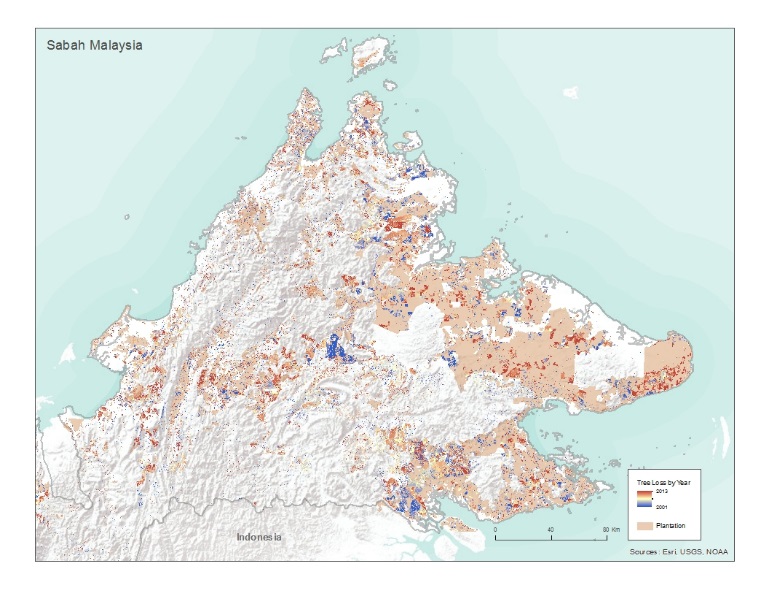


Figure S2. Mapped plantations and tree cover loss in Malaysia between 2001-2013 for (a) peninsular Malaysia, (b) Sabah and (c) Sarawak. Early years of loss in the time series are shown in blue and later years of loss shown in red.

**References**

Baccini, A. et al. Estimated carbon dioxide emissions from tropical deforestation improved

by carbon-density maps. Nature Clim. Change 2, 182–185 (2012). URL <http://dx>. [doi.org/10.1038/nclimate1354](http://doi.org/10.1038/nclimate1354).

Breiman, L. Random forests. Machine learning 45, 5–32 (2001).

Chave, J. et al. Tree allometry and improved estimation of carbon stocks and balance in

tropical forests. Oecologia 145, 87–99 (2005).

Hansen, M. C. et al. High-Resolution Global Maps of 21st-Century Forest Cover Change.

Science 342, 850–853 (2013).

Hijmans, R. J., Cameron, S. E., Parra, J. L., Jones, P. G. & Jarvis, A. Very high resolution interpolated climate surfaces for global land areas. International Journal of Climatology 25, 1965–1978 (2005).

Schaaf, C. et al. First operational brdf, albedo nadir reflectance products from modis.

Remote Sensing of Environment 83, 135–148 (2002).

1. Indonesia is not included in Figure S1(d) although it did sign the Declaration; nor is Brazil, which did not. [↑](#footnote-ref-1)
